# Fracture of the Femoral Component Caused by Insufficient Crimping after Modern Unicompartmental Knee Arthroplasty

**DOI:** 10.1155/2019/5938598

**Published:** 2019-12-03

**Authors:** Yoichi Ishibashi, Tasuku Mashiba, Masaki Mori, Tetsuji Yamamoto

**Affiliations:** Department of Orthopedic Surgery, Kagawa University Faculty of Medicine, Kagawa, Japan

## Abstract

Fracture of a femoral component after modern unicompartmental knee arthroplasty is very rare. Although this is not the first case on this subject, no study has reported insufficient crimping as the cause of femoral component loosening that led to breakage of a metallic component. A 69-year-old man underwent medial unicompartmental knee arthroplasty for right medial knee osteoarthritis. His early postoperative course was good; however, the 1-year postoperative radiograph showed an apparent radiolucent line around the femoral component, and he occasionally had right knee pain. However, he had been followed up conservatively because he had been doing well even while doing heavy agricultural work. At 8 years after surgery, because breakage of the femoral component was found, revision surgery was performed using bicruciate-retaining total knee arthroplasty. The removed fractured femoral component revealed a thick cement mantle detached from the bone surface. The postoperative course of the patient after the revision surgery was excellent. We suggest that the causes of femoral component breakage include early implant loosening caused by uneven cement crimping of the femoral component to the bone and excessive loading stress as a result of heavy labour.

## 1. Introduction

Unicompartmental knee arthroplasty (UKA) was used for the first time in the 1970s for patients with single compartment osteoarthritis [1]. Compared to total knee arthroplasty (TKA), UKA is less invasive and has greater advantages, such as a good range of motion, quick recovery, and similarity to a natural knee joint [2, 3]. However, UKA is associated with lower survival rates than TKA [4]. Several studies have reported causes of UKA failure, such as aseptic loosening, osteoarthritis progression, polyethylene wear, infection, instability, and bone fracture [5, 6]. Fracture of the metallic components is a rare complication after UKA; in particular, UKAs performed after 2000 have shown very low incidence of such fractures [7]. Here, we report a case of a fractured femoral component after UKA caused by loosening of the implant probably due to insufficient crimping of the femoral component. Although this is not the first case on this subject, to the best of our knowledge, there is no published report on insufficient crimping as the cause of femoral component loosening that led to fracture of a metallic component after modern UKA.

## 2. Case Presentation

A 69-year-old man presented with a 10-year history of right knee pain due to medial osteoarthritis. He worked as an agriculturist, and his weight and body mass index (BMI) were 70 kg and 27.6 kg/m^2^, respectively.

**Figure 1:**
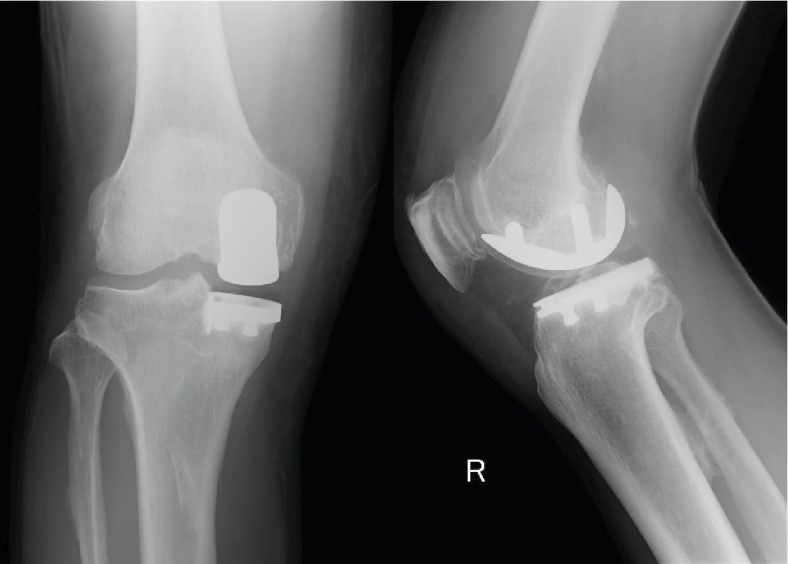
Immediate postoperative radiography demonstrating good implant position and alignment.

His range of motion of his right knee was 5°–135°, and the collateral and cruciate ligaments were intact. Preoperative radiography demonstrated the presence of medial osteoarthritis. Medial UKA (using the Zimmer Unicompartmental High Flex Knee System; Zimmer, Warsaw, IN, USA) was performed because his symptoms did not improve with conservative treatment. Surgery was performed via the tibia-dependent cut technique using a spacer block, and cement-retained prostheses were implanted. A femoral component of size E, a tibial component of size 3, and an 11 mm polyethylene insert were placed. No complications were observed during the perioperative period. The postoperative knee range of motion was 0°–140°, and no knee instabilities were noted. No abnormalities and problems with the positioning of the component were noted on the postoperative radiograph (Figure 1).

**Figure 2:**
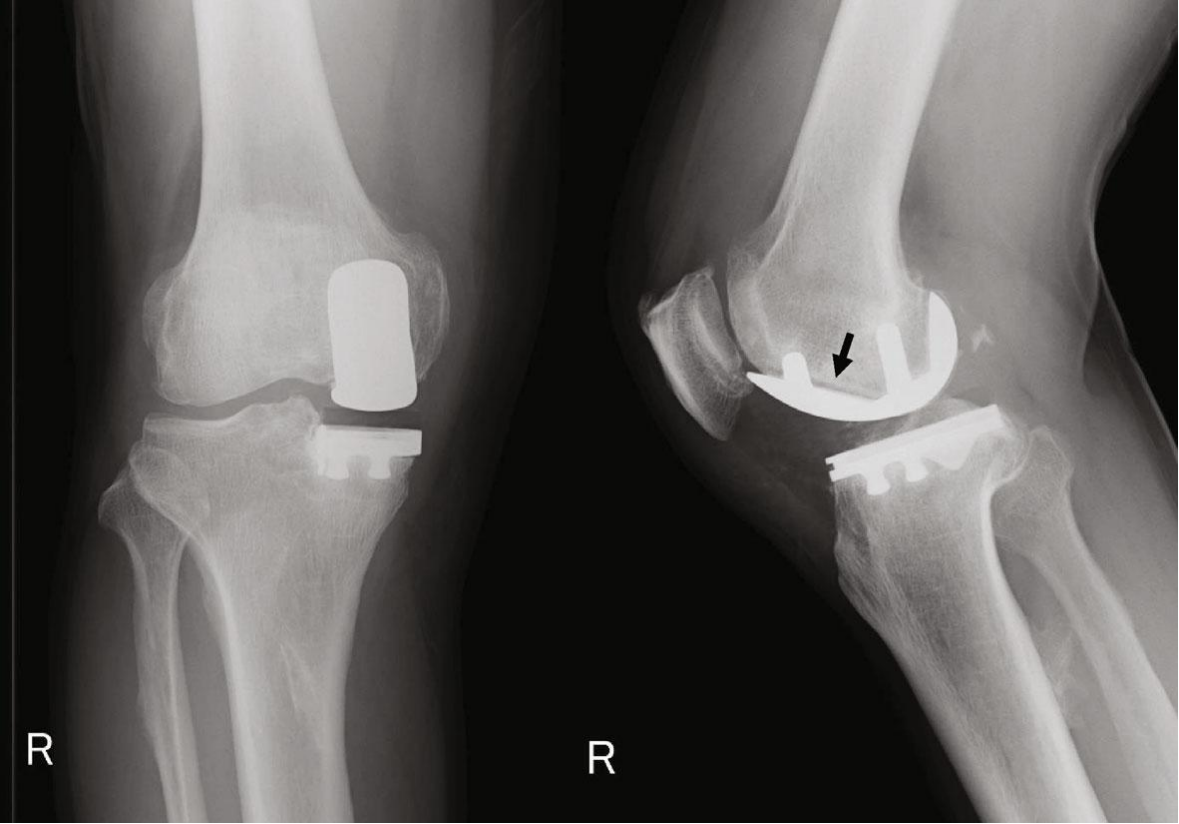
Plain radiography demonstrating a radiolucent line around the femoral component at the 1-year follow-up.

At the 1-year postoperative follow-up, the patient reported occasional right knee pain. Radiography revealed a radiolucent line around the femoral component (Figure 2). However, he was followed up by observation alone because he only had occasional mild right knee pain that was relieved by medication.

**Figure 3:**
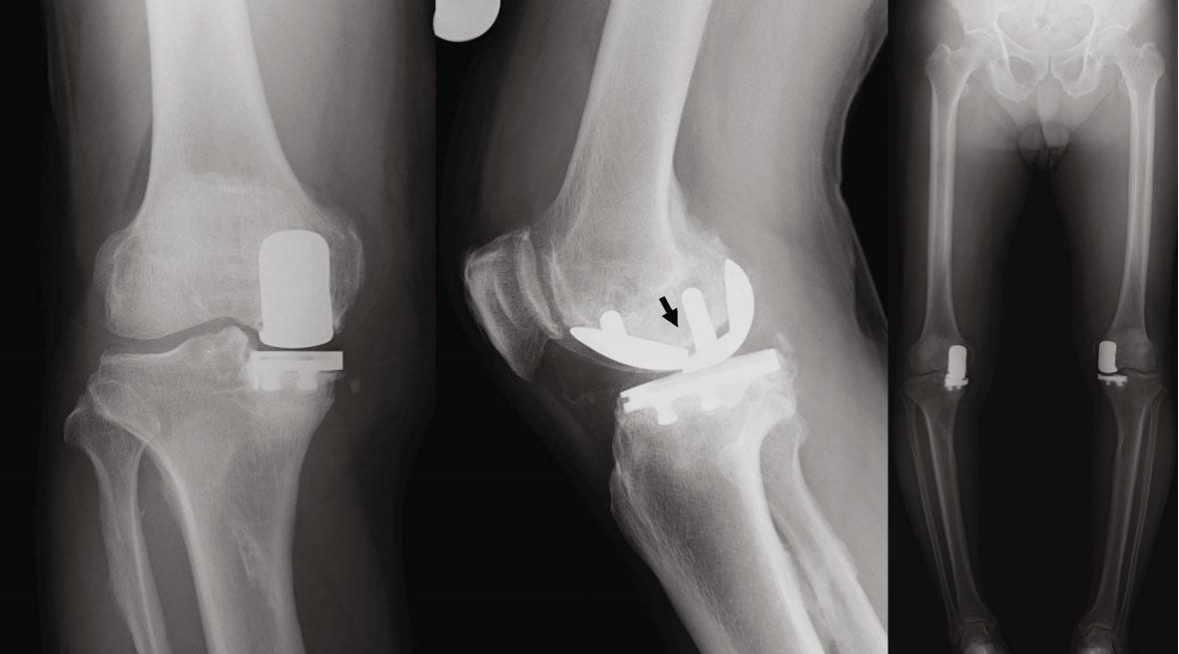
Radiography demonstrating fracture and loosening of the femoral component at the 8-year follow-up. Marked narrowing of the medial joint space is also seen.

**Figure 4:**
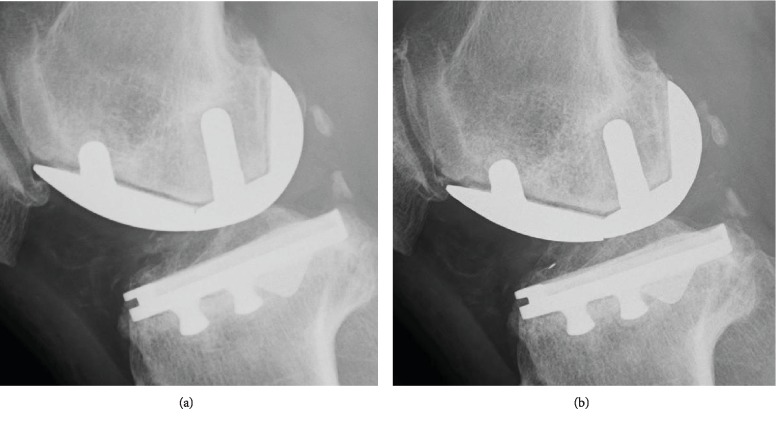
A small crack sign is found in the femoral component, which had been missed at the (a) 6- and (b) 7-year postoperative follow-up.

At the 8-year postoperative follow-up, he complained of increasing right knee pain, and radiography revealed fracture of a metallic femoral component, loosening of the femoral component, and marked narrowing of the medial joint space (Figure 3). There were no trauma events postoperatively. On careful analysis of past radiographs, a small crack sign was found in the femoral component, which had been missed at the 6- and 7-year postoperative follow-up (Figure 4). However, his symptoms were not severe even at 8 years postoperatively. His right knee was not swollen, and the range of motion was well preserved. He could walk approximately 10 km and also do farm work. Although unresurfaced compartments were well-preserved, because the varus knee deformity was >10° and uncorrectable, bicruciate-retaining (BCR) TKA revision surgery was planned instead of revision UKA to preserve the remaining anterior cruciate ligament.

**Figure 5:**
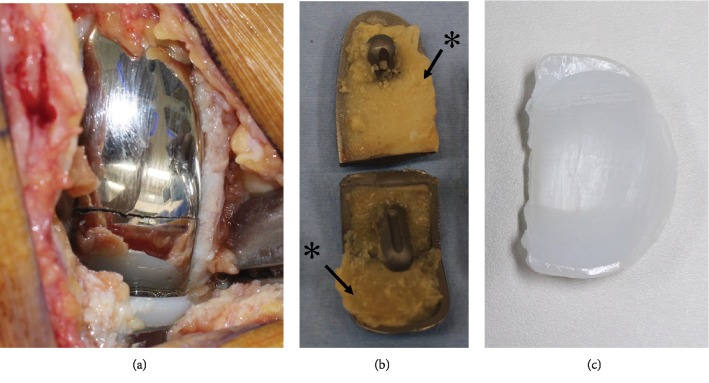
(a) Fracture of the femoral component is noted intraoperatively. (b) Thick cement mantle observed at both lateral sides of the distal surface and at the medial side of the posterior surface (^∗^). (c) Marked wear of the middle part of the polyethylene insert.

**Figure 6:**
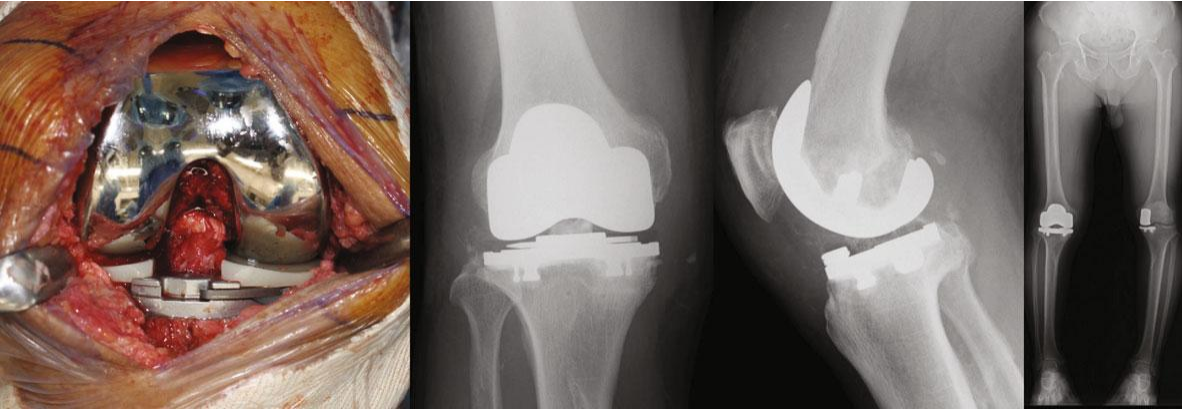
Revision surgery using cemented bicruciate-retaining total knee arthroplasty.

Intraoperatively, a fracture and loosening of the femoral component were observed (Figures 5(a) and 5(b)). There was marked wear of the middle part of the polyethylene insert (Figure 5(c)). However, the tibial component was well-fixed. Clear joint fluid and mild synovitis were detected. There was no sign indicating infection during the surgery, and all culture examinations were negative. The posterior and anterior cruciate ligaments were stable, and there was sufficient bone stock after the removal of the UKA implants. Therefore, we performed revision surgery using cemented BCR-TKA (Vanguard XP Total Knee System; Zimmer Biomet, Warsaw, IN, USA) based on the desire to preserve intact cruciate ligaments (Figure 6). No complications were noted after the revision surgery. At the 1-year follow-up after the revision surgery, the patient reported no pain and could walk 10 km and return to farm work. His right knee range of motion was 0°–130°.

Informed consent was obtained from the patient, and this case study was approved by the concerned institutional review board.

## 3. Discussion

Fracture of the metallic components after UKA is a rare complication. The Swedish Knee Registry reported that fracture of components after UKA occurred in 24 out of 1135 cases (2%), but detailed information, such as the parts involved and specific causes, were not described [8]. Manzotti et al. reported the incidence of fracture of the metallic components after UKA as 4.9% of all UKA failures, and the cause of this high incidence was reported to be the regular reporting of complications in their centre [9]. Gilg et al. reported that the rate of fracture of femoral and tibial components after UKA was 0.85% in three clinical studies [7]. However, there are no additional reports of a high incidence rate, and the exact incidence rate is unknown. More than 1000 UKAs have been performed in our facility; however, this is the first reported case of fracture of a metallic component.

Most previous reports of fracture of the metallic components in the literature occurred in the femoral component [9]. There is only one report of fracture of a tibial metallic component [9]. Additionally, fractures of the femoral component are likely to occur close to the attached peg [9, 10]. Several previous reports have described that the cause of fractures of the femoral component is loosening of the implant induced by osteolysis of the femoral condyle secondary to polyethylene wear debris [11–13]. Fatigue fracture caused by repeated stress of the femoral component has also been reported as a cause [12]. Some reports have suggested that the older model of UKA implants had a high failure rate and that increasing the metal implant thickness and strengthening the pegs in the femoral component can decrease the risk of fracture of the femoral component [10, 14]. A BMI of >30 and a fixed bearing insert were also reported as risk factors for component fracture [9].

In the present case, the fracture site was the boundary between the distal and oblique posterior surfaces of the femoral component, which is the thinnest part of the femoral component. In addition, the metallic fracture site was, to some extent, distant from a peg. This is in contrast to a finding by Manzotti et al. [9] in which fracture of the femoral component occurred adjacent to the peg in old generation UKA implants, such as UC-plus, St George Sled, or Allegretto prosthesis. These authors, along with some other researchers [10, 14], have suggested that older UKA femoral implant designs have a higher risk of breakage and that this risk has reduced with newer UKA designs that incorporate a thicker prosthesis and/or reinforcement at the fixation pegs. The model used in the present case was a modern UKA implant with a better implant design and improved implant material compared to those of older UKA prostheses; however, it is likely that a fatigue fracture can occur at the weakest part of the femoral component in case of implant loosening. In our case, the removed femoral component revealed a thick cement mantle at the lateral part of the distal surface and the medial part of the posterior surface (Figure 5(b)), indicating that insufficient contact between the femoral component and the cutting bone surface at the initial surgery led to early loosening of the implant. Furthermore, this patient continued with his daily heavy farm work, including carrying 30 kg rice bales. This consequent repetitive overloading on the unstable loosened femoral component might have caused a fatigue metallic fracture. To our knowledge, this is the first case report suggesting that insufficient crimping of the femoral component with cement can cause fracture of a femoral component after modern fixed-bearing UKA.

## 4. Conclusion

Fracture of the metallic femoral component after UKA is a rare complication associated with modern UKA prostheses. The present report indicates that implant loosening due to insufficient crimping of the femoral component using cement and excessive loading can cause fracture of a femoral component even with a modern implant with a better design. Thus, a careful procedure is necessary at the time of implantation with cement.
